# The effect of a regional care model on cardiac catheterization rates in patients with Acute Coronary Syndromes

**DOI:** 10.1186/s12913-014-0550-0

**Published:** 2014-11-08

**Authors:** Helen J Curran, Jaroslav Hubacek, Danielle Southern, Diane Galbraith, Merril L Knudtson, William A Ghali, Michelle M Graham, on behalf of the Alberta Provincial Project for Outcome Assessment in Coronary Heart Disease (APPROACH) Investigators

**Affiliations:** Division of Cardiology, Dalhousie University, room 2145, Halifax Infirmary, 1796 Summer Street, Halifax, B3H 3A7 Nova Scotia Canada; The New Brunswick Heart Center, Saint John, New Brunswick Canada; Centre for Health and Policy Studies University of Calgary, Calgary, Alberta Canada; The APPROACH Project Office, University of Calgary, Calgary, Alberta Canada; Libin Cardiovascular Institute, University of Calgary, Calgary, Alberta Canada; Department of Medicine and Mazankowski Alberta Heart Institute, University of Alberta, Edmonton, Alberta Canada; Division of Cardiology, University of Alberta Hospital, 8440-112 Street, Edmonton, T6G 2R7 Alberta Canada

**Keywords:** Acute coronary syndrome, Catheterization, Mortality, Registries, Regional care model

## Abstract

**Background:**

Patients with ACS often present to community hospitals without on-site cardiac catheterization and revascularization therapies. Transfer to specialized cardiac procedural centers is necessary to provide access to these procedures. We evaluated process of care within a regional care model by comparing cardiac catheterization and revascularization rates and outcomes in ACS patients presenting to community and interventional hospitals.

**Methods:**

We evaluated a total of 6154 patients with ACS admitted to Southern Alberta hospitals (where a distinct regional care model for ACS exists) between January 1, 2005 and December 31, 2009. We compared cardiac catheterization and revascularization rates during index hospitalization among patients admitted to community and interventional hospitals. Thirty day and 1-year survival were also evaluated.

**Results:**

Catheterization was performed more often in patients presenting to community hospitals compared to the interventional facility (respectively 69.5% and 51.4%, p < 0.0001). Catheterization within 72 hours of admission occurred in 48% of patients presenting to the interventional center and in 68.3% of community patients (P < 0.0001). In patients undergoing catheterization, revascularization (PCI and/or CABG) was also performed more frequently in the community group (74.5% vs 56.1%, P < 0.0001). Risk adjusted mortality rates were the same for patients undergoing cardiac catheterization regardless of hospital of initial presentation.

**Conclusion:**

ACS patients presenting to community centers associated with a regional care model had effective access to cardiac catheterization and revascularization. These findings support the importance of regional initiatives and processes of care that facilitate access to cardiac catheterization for all ACS patients.

## Background

Acute coronary syndromes (ACS) represent a common cause for hospital admissions and mortality in patients with cardiovascular disease. In patients with non-ST elevation myocardial infarction (NSTEMI), an early invasive strategy, in patients without contraindications or prohibitive comorbidities, is superior to a selective invasive strategy in reducing rehospitalization and myocardial infarction (MI) [1,2]. Indeed, guidelines endorse an early invasive strategy defined as cardiac catheterization and revascularization within 48–72 hours of presentation for high-risk patients [3–6]. However, patients with ACS usually present to the nearest acute care facilities for evaluation and management. These are often community hospitals that lack cardiac catheterization and revascularization (percutaneous coronary intervention [PCI] and coronary artery bypass grafting [CABG]) abilities. Efficient inter-hospital transfer between community and tertiary centers is therefore necessary to provide access to these procedures. Previous studies have demonstrated that a significant number of ACS patients in the community do not get transferred to interventional capable centers for invasive management [7–9].

We have developed a large, population-based clinical registry that captures all patients undergoing cardiac catheterization and revascularization in Alberta, Canada since 1995. The subsequent expansion of this registry to include all cardiac admissions in Southern Alberta provides a unique opportunity to examine practice patterns (such as transfer for cardiac catheterization) and outcomes in unselected ACS patients. The purpose of this study was to evaluate process of care within a regional care model by comparing cardiac catheterization and revascularization rates and outcomes in ACS patients presenting to community and interventional hospitals.

## Methods

All data were derived from the Alberta Provincial Program for Outcome Assessment in Coronary Heart disease (APPROACH). APPROACH is an ongoing prospective cohort study of all Alberta residents undergoing cardiac catheterization for coronary artery disease since 1995, which expanded in 2004 to include cardiac admissions in Southern Alberta. The initiative has been previously described [10]. In brief, this population-based, multiple-year inception cohort database contains detailed information on socio-demographic characteristics, presence of risk factors and comorbidities, disease-specific variables, coronary catheterization results, post-catheterization referral decisions, records of actual revascularization and long-term outcomes including survival and quality of life. Data from APPROACH are routinely enhanced by merging the clinical registry data to administrative records to supplement clinical information available on all patients. This data enhancement methodology has been validated and previously reported [11]. Patient survival from catheterization and/or revascularization until death is ascertained through semi-annual linkage to Alberta Vital Statistics records. The APPROACH registry has an approved privacy impact assessment. The University of Calgary and University of Alberta Research Ethics Boards have approved APPROACH registry data collection and linkages with secondary sources.

For the present study we identified all patients over the age of 18 with ACS (NSTEMI and unstable angina (UA)) admitted to hospitals in Southern Alberta. This region is a large geographic area of over 1.6 million people served by one interventional center in the city of Calgary with a constellation of smaller community hospitals in surrounding areas. This Calgary Health Region is one of the largest, fully integrated health care systems in Canada.

Patients were categorized according to location and facilities available in the hospital of initial admission: 1) community hospitals: comprised of secondary referral centers staffed by specialists and/or general practitioners and smaller primary centers without on-site specialty physicians. All community centers lack on-site cardiac catheterization facilities and are located a minimum of 49 kilometers (km) and a maximum of 291 km (average 220 km) from the interventional center, 2) interventional hospital: the only tertiary care center with on-site cardiac catheterization and revascularization abilities and cardiology specialists. Each rural region has independent ACS management protocols, however, when a patient requiring cardiac catheterization is identified, referring physicians complete a standard referral form that is sent to the catheterization center by fax, along with relevant history, GRACE score, laboratory data, and other tests if applicable. When the referral is complete, it is reviewed by a member of the interventional cardiology group for approval and triage for urgency and arrangements for transport to the interventional center are made.

### Statistical analysis

The primary outcomes of interest were cardiac catheterization and revascularization rates during index admission among patients admitted to community versus interventional hospitals. Baseline comparisons of clinical characteristics between patient groups were made by the Chi Square Test for categorical variables and by ANOVA for continuous variables. Kaplan-Meier analysis was used to present the unadjusted thirty-day and one-year survival rates from the index hospitalization for each patient group. Cox proportional hazard models were then used to calculate survival following risk adjustment for the clinical characteristics and comorbidities presented in Table 1. All analyses were conducted using SAS 9.2, (Cary, North Carolina).

**Table 1:** **Baseline characteristics**

**Characteristic**	**Interventional N =3562**	**Community N =2592**	**p value**
Age (years)	66.4 ± 12.6	66.3 ± 13.0	0.84
Male	2474 (69.5%)	1768 (68.2%)	0.31
Diabetes	872 (24.5%)	678 (26.2%)	0.14
Hypertension	2357 (66.2%)	1732 (66.8%)	0.59
Dyslipidemia	2636 (74.0%)	1999 (77.1%)	0.005
Smoker	675 (19.0%)	660 (25.5%)	<0.0001
Previous MI	959 (26.9%)	677 (26.2%)	0.50
Previous PCI	880 (24.7%)	484 (18.7%)	<0.0001
Previous CABG	413 (11.6%)	227 (8.8%)	0.0003
Previous heart failure	252 (7.1%)	238 (9.2%)	0.003
Chronic renal failure	115 (3.2%)	74 (2.9%)	0.40
Acute renal failure	10 (0.3%)	10 (0.4%)	0.48
Dialysis	76 (2.1%)	29 (1.1%)	0.002
Cerebrovascular disease	292 (8.2%)	196 (7.6%)	0.36
Peripheral vascular disease	172 (4.8%)	137 (5.2%)	0.46
Mean heart rate (beats/min)	75.5 (19.3)	76.5 (20.2)	0.05
Mean systolic BP (mmHg)	138.8 (25.7)	142.1 (27.4)	<0.0001
Mean diastolic BP (mmHg)	77.8 (16.2)	80.2 (17.4)	<0.0001
Systolic BP < 100	136 (3.9%)	106 (4.2%)	0.63
Presence of shock	32 (0.9%)	18 (0.7%)	0.38
ECG dynamic changes	419 (11.8%)	437 (16.9%)	<0.0001
Presentation diagnosis			<0.0001
Unstable angina	2286 (42.5)	1334 (33.7)	
NSTEMI	1276 (23.7)	1259 (31.7)	
Non-invasive tests^†^ performed within 3 months prior to admission	471 (13.2%)	288 (11.1%)	0.013
Catheterization not received	1730 (48.6%)	1834 (32.2%)	<0.0001
Catheterization received	11832 (51.4%)	1759 (67.8%)	
Within 24 hours of admission	594 (16.7%)	255 (9.8%)	<0.0001
Within 48 hours of admission	995 (54.3%)	571 (32.5%)	<0.0001
Within 72 hours of admission	1260 (68.8%)	844 (48%)	<0.0001

Values are expressed as mean ± SD and number (%).

*MI* = Myocardial infarction, *PCI* = Percutaneous coronary intervention, *CABG* = Coronary artery bypass grafting, *BP* = Blood pressure, *ECG* = Electrocardiogram, *mmHg* = Millimeters of mercury, *NSTEMI* = Non-ST elevation myocardial infarction.

^†^Non-invasive tests includes nuclear thallium, stress ECHO, treadmill, CT, MRI and peripheral angiography.

## Results

Our cohort consisted of 6154 ACS patients admitted to hospitals in Southern Alberta between January 1, 2005 and December 31, 2009. Of these patients, 2592 (42.1%) were admitted initially to community hospitals and 3562 (57.9%) to the interventional center. Baseline patient characteristics were analyzed according to type of admitting hospital (Table 1) and cardiac catheterization status (Table 2). Smoking, dyslipidemia, previous heart failure, initial diagnosis of NSTEMI and the presence of dynamic electrocardiogram changes were all more prevalent in patients admitted to community hospitals. Patients admitted to the interventional hospital more commonly had a history of previous revascularization (PCI /CABG) and were more likely to be on dialysis than those admitted to community hospitals.

**Table 2:** **Baseline characteristics and cardiac catheterization status**

	**Interventional**			**Community**			
**Characteristic**	**No cath N = 1,730**	**Cath N = 1,832**	**P-value**	**No cath N = 791**	**Cath N = 1,801**	**P-value**	**Overall P-value**
Age (years)	68.1 (13.2)	64.8 (11.9)	<0.0001	69.6 (14.6)	64.9 (12.0)	<0.0001	<0.0001
Male	1,147 (66.3%)	1,327 (72.4%)	<0.0001	472 (59.7%)	1,296 (72.0%)	<0.0001	<0.0001
Diabetes	441 (25.5%)	431 (23.5%)	0.173	216 (27.3%)	462 (25.7%)	0.377	0.18
Hypertension	1,157 (66.9%)	1,200 (65.5%)	0.386	511 (64.6%)	1,221 (67.8%)	0.112	0.32
Dyslipidemia	1,227 (70.9%)	1,409 (76.9%)	<0.0001	513 (64.9%)	1,486 (82.5%)	<0.0001	<0.0001
Smoker	310 (17.9%)	365 (19.9%)	0.127	157 (19.9%)	503 (27.9%)	<0.0001	<0.0001
Previous MI	496 (28.7%)	463 (25.3%)	0.022	246 (31.1%)	431 (23.9%)	0.0001	0.0002
Previous PCI	449 (26.0%)	431 (25.3%)	0.093	140 (17.7%)	344 (19.1%)	0.399	<0.0001
Previous CABG	235 (13.6%)	178 (9.7%)	0.0003	91 (11.5%)	136 (7.6%)	0.001	<0.0001
Previous heart failure	162 (9.4%)	90 (4.9%)	<0.0001	123 (15.6%)	115 (6.4%)	<0.0001	<0.0001
Chronic renal failure	66 (3.8%)	49 (2.7%)	0.054	27 (3.4%)	47 (2.6%)	0.258	0.12
Acute renal failure	7 (0.4%)	3 (0.2%)	0.175	8 (1.0%)	2 (0.1%)	0.0007	0.001
Dialysis	43 (2.5%)	33 (1.8%)	0.158	10 (1.3%)	19 (1.1%)	0.641	0.008
Cerebrovascular disease	149 (8.6%)	143 (7.8%)	0.380	79 (10.0%)	117 (6.5%)	0.002	0.013
Peripheral vascular disease	89 (5.1%)	83 (4.5%)	0.393	53 (6.7%)	83 (4.6%)	0.028	0.097
Mean heart rate (beats/min)	74.6 (19.8)	76.3 (18.9)	0.012	79.2 (20.6)	75.4 (20.0)	<0.0001	<0.0001
Mean systolic BP (mmHg)	136.8 (25.7)	140.7 (25.6)	<0.0001	140.7 (27.8)	142.7 (27.2)	0.092	<0.0001
Mean diastolic BP (mmHg)	75.7 (15.9)	79.8 (16.2)	<0.0001	78.0 (17.0)	81.2 (17.5)	<0.0001	<0.0001
Systolic BP < 100	78 (4.7)	58 (3.2)	0.029	39 (5.1)	67 (3.8)	0.142	0.068
Presence of shock	13 (0.8%)	19 (1.0%)	0.367	9 (1.1%)	9 (0.5%)	0.072	0.22
ECG dynamic changes	189 (10.9%)	230 (12.6%)	0.131	138 (17.5%)	299 (16.6%)	0.597	<0.0001
Presentation diagnosis			<0.0001			<0.0001	<0.0001
Unstable angina	523 (30.2%)	753 (41.1%)		306 (38.7%)	953 (52.9%)		
NSTEMI	1,207 (69.8%)	1,079 (58.9%)		485 (61.3%)	848 (47.1%)		
Non-invasive tests^†^ performed within 3 month prior to admission	360 (20.8%)	256 (14.0%)	<0.0001	100 (12.6%)	248 (13.8%)	0.438	<0.0001

Values are expressed as mean ± SD and number (%).

*MI* = Myocardial infarction, *PCI* = Percutaneous coronary intervention, *CABG* = Coronary artery bypass grafting, *BP* = Blood pressure, *ECG* = Electrocardiogram, *mmHg* = Millimeters of mercury, *NSTEMI* = Non-ST elevation myocardial infarction.

^†^Non-invasive tests includes nuclear thallium, stress ECHO, treadmill, CT, MRI and peripheral angiography.

**Figure 1:**
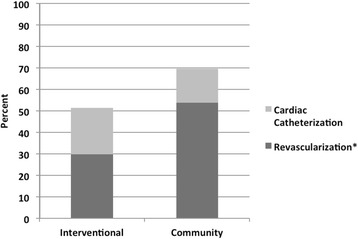
**Cardiac catheterization and revascularization rates by presenting hospital (Revascularization defined as percutaneous coronary intervention or coronary artery bypass grafting).** Catheterization: Int 1,832 (51.4%) vs. comm 1,801 (69.5%), P<0.0001.Revascularization: Int 1,027 (56.1%) vs. comm 1,342 (74.5%), P<0.0.

Figure 1 depicts cardiac catheterization and revascularization rates according to type of admitting hospital. Catheterization was performed more frequently in patients initially admitted to community hospitals than those admitted initially to the interventional facility (respectively 69.5% and 51.4%, p < 0.0001). In patients undergoing catheterization, revascularization (PCI and/or CABG) was also performed more frequently in the community group compared to the interventional group (respectively, 74.5% and 56.1%, P < 0.0001). The overall mean time to cardiac catheterization was 3.2 days. Patients admitted to the interventional center underwent catheterization sooner than those admitted to community centers (2.6 vs. 4 days, p < 0.001). In the interventional group, 9.8% underwent catheterization within 24 hours of admission compared to 16.7% in the community group. Within 48 hours from admission, 32.5% and 54.3% of the interventional and community patients underwent catheterization and within 72 hours, 48% and 68.3% had undergone the procedure (p < 0.0001).

**Figure 2:**
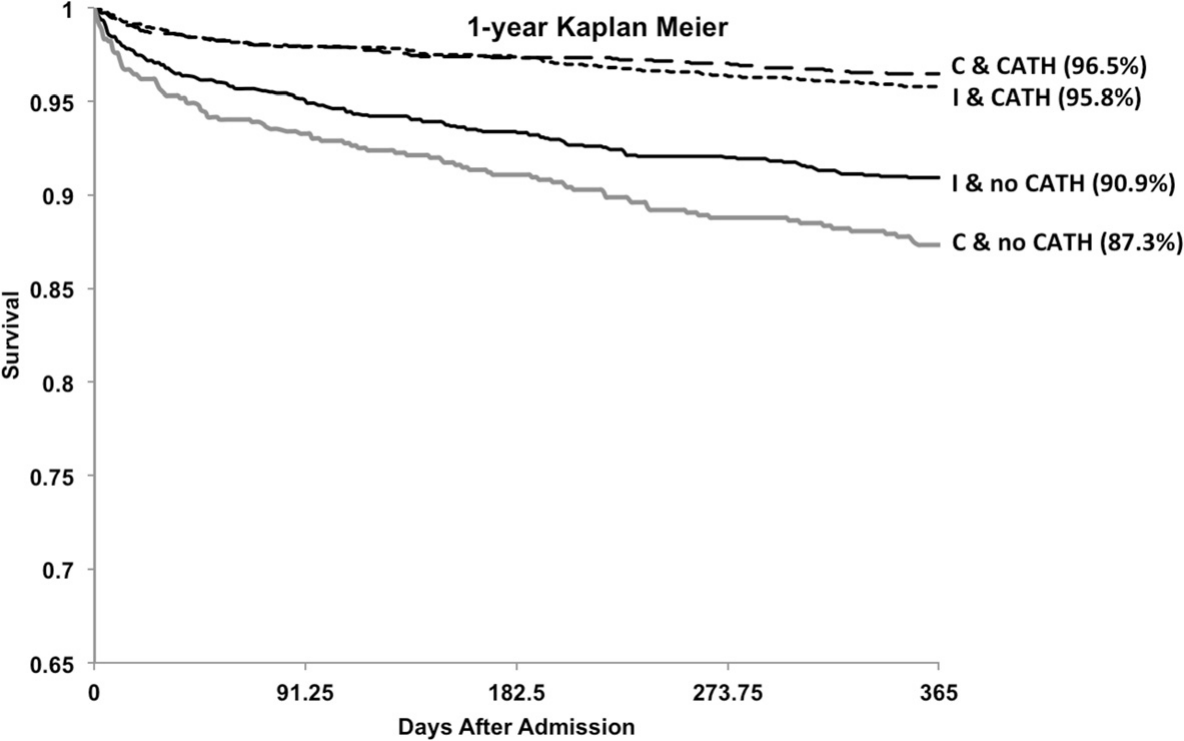
**Kaplan Meier plot demonstrating survival from admission with acute coronary syndrome stratified by cardiac catheterization status (C = community hospitals, M = metropolitan hospitals, I = interventional hospital, cath = cardiac catheterization).**

Figure 2 demonstrates Kaplan Meier survival curves extending to one year of follow up. There was an early separation of the curves that persisted with longer follow-up, with crude mortality rates significantly lower in patients undergoing cardiac catheterization in both community (12.7% vs. 3.5%) and interventional (9.1% vs. 4.2%) centers p < 0.0001. Survival was lower for patients not referred for catheterization from an admitting community hospital compared to patients first admitted to the interventional center but not referred for catheterization (87.3% vs. 90.9%, p = 0.003).

Table 3 demonstrates crude and adjusted hazard ratios for our cohort. After adjusting for baseline risk factor characteristics seen in Table 1, no difference in outcome at 30 days or 1 year was noted between those admitted to the interventional center and those initially admitted to community centers.

**Table 3:** **Hazard ratios for 30-day and 1-year mortality**

**Cardiac cath status**	**Presenting hospital**	**30-day crude HR (95% CI)**	**30-day adjusted HR (95% CI)**	**1-year crude HR (95% CI)**	**1-year adjusted HR (95% CI)**
Cath	Interventional	**Reference**	**Reference**	**Reference**	**Reference**
Cath	Community	1.16 (0.65, 2.05)	1.04 (0.58, 1.85)	0.84 (0.60, 1.18)	0.79 (0.56, 1.11)
No Cath	Interventional	2.58 (1.57, 4.24)	2.38 (1.43, 3.97)	2.23 (1.69, 2.93)	2.05 (1.55, 2.72)
No Cath	Community	3.86 (2.27, 6.56)	2.31 (1.32, 4.06)	3.15 (2.33, 4.26)	2.06 (1.50, 2.84)

## Discussion

This analysis from a large cohort of geographically diverse patients indicates that regionalization of tertiary ACS services enables access to cardiac catheterization for patients from community centers. Indeed, within this regional care model, patients presenting initially to community hospitals actually had higher rates of cardiac catheterization and revascularization than those presenting initially to the interventional center, and underwent these procedures in a timely fashion.

The advantages of regional care centers for the treatment of ST elevation myocardial infarction (STEMI) have been clearly described [12–14]. Regional approaches to STEMI care focus on rapid identification and early reperfusion therapy with the goal of reducing morbidity and mortality. The essential components of regional STEMI care plans include detailed treatment and transfer protocols, community planning, resources, education, quality improvement analysis and research [15]. Likewise, the development of regionalized comprehensive stroke centers for the care of patients with acute stroke has addressed disparities in the delivery of stroke services particularly between urban and rural centers [16–18].

Few studies have evaluated the regionalization of care for patients with NSTEMI, particularly in the geographically robust provinces of Canada. The Heart Protection Partnership (HPP) project was an Australian initiative developed to audit compliance with evidence-based treatments in patients with acute coronary syndromes treated at interventional and non-interventional centers across the country. The program identified treatment gaps, particularly in non-interventional centers, and provided feedback to individual centers with the purpose of improving compliance with benchmark standards of care [19]. Ideally, regional centers for ACS should provide effective and efficient care for patients by improving access to cardiac catheterization, revascularization, specialist physicians and to new technologies or medications not available at other centers. The regional care model within Southern Alberta consists of one PCI capable center that provides specialized procedural cardiac care to numerous smaller and geographically distant community centers. This model’s process of care involves a well established referral system for cardiac catheterization whereby direct communication between community and subspecialty physicians, delivery of detailed patient information and efficient risk based triage of community ACS patients translates into timely referral for and access to cardiac catheterization.

Geographic disparity in cardiac catheterization and revascularization rates in ACS are well described. A large registry study in Denmark found higher coronary angiography and revascularization rates in ACS patients residing close to an interventional facility compared to those residing at a further distance, with a higher probability of receiving catheterization and revascularization if admitted directly to an interventional center [8]. The Dartmouth Atlas of Cardiovascular Health Care in the United States recognized that rates of cardiac catheterization varied substantially from the national average in many referral regions [20]. Another group of investigators from the United States demonstrated that less than half of patients presenting to community hospitals with ACS were transferred to tertiary hospitals for cardiac catheterization [9]. This geographical variance has also been described in Canada where investigators in the province of Nova Scotia found lower rates of cardiac catheterization for patients residing outside of a metropolitan area [21]. Data from the New Zealand Cardiac Society Audit Group demonstrated that patients with ACS admitted to non-interventional centers were less likely to be referred and had longer time delays to access cardiac catheterization compared to those admitted to interventional centers [22]. In our study we found that despite large geographical barriers of up to 291 Km, patients initially presenting to community hospitals had similar or even greater access to cardiac catheterization compared to those initially presenting to the interventional facility.

Randomized controlled trials and meta-analyses have demonstrated better clinical outcomes in NSTEMI patients treated with an early invasive approach (early cardiac catheterization and revascularization as required for moderate to high risk NSTEMI) [22–26]. These studies showed a consistent reduction in recurrent ischemia and re-infarction but inconsistencies in the survival benefit. In our study we found better 30-day and 1-year outcomes in patients managed invasively compared to those managed conservatively. Due to the observational nature of this study, selection bias may influence outcomes and partially account for baseline differences in patients chosen for cardiac catheterization. This limits generalization of our findings to different populations. Further evaluation of detailed patient characteristics and comorbidities, patient preferences, the shared decision-making process and potential referral physician selection bias are necessary to explain these findings.

In this study, there were no differences in 30-day or 1-year risk adjusted mortality between patients admitted to interventional and community hospitals, despite the differences noted in cardiac catheterization and revascularization rates. Differences in adherence to acute and long- term, guideline based medical therapies, in addition to numerous other patient care factors not evaluated in this study, could account for these findings. Similar findings were noted in a study evaluating outcomes of NSTEMI patients treated in academic and non-academic centers in the United States. Despite higher utilization of guideline based medications, cardiac catheterization and revascularization in academic centers, there were no differences in 1-year mortality compared to non-academic center patients [27].

Our findings support the development of regional initiatives and processes of care that ensure appropriate access to cardiac catheterization for patients with ACS. Facilitating transfer of patients from hospitals without cardiac catheterization capabilities to regional invasive centers is essential for favorable outcomes in ACS. Such a regional care model successfully exists in Southern Alberta where management practices of non-cardiologists in community centers encompass a strong compliance with evidence-based, regional cardiac catheterization referral guidelines and protocols and an aggressive transfer strategy for invasive investigation is the predominant model of care in community centers. Performance of catheterization within guideline recommended timeframes for all patients is a recognized area for improvement in this regional model. The development of formal repatriation processes of care to ensure availability of catheterization facilities may help to achieve these goals.

This study has limitations. We evaluated cardiac catheterization rates for ACS patients in a Canadian regional model that may differ in structure from other international models; nonetheless, our findings provide insights into important ACS management concepts. Although we demonstrated good clinical outcomes in patients undergoing cardiac catheterization we were unable to determine why some patients were not referred for cardiac catheterization or whether the treatment of conservatively managed patients was optimal. Additionally, our registry does not audit against key performance indicators or provide detailed information regarding guideline based medical therapies. Admission to presenting hospitals was not randomly assigned, and consequently our results may be confounded by other unmeasured factors. However, these limitations are balanced by the strength of our evaluation of a large, unselected group of real-world ACS patients.

## Conclusion

In this large, contemporary, registry-based study we describe a successful regional care model in which ACS patients first admitted to community hospitals have effective access to cardiac catheterization despite geographic and available services barriers. These findings support the importance of regional initiatives and processes of care that facilitate access to cardiac catheterization for ACS patients.

## Abbreviations

ACS: Acute coronary syndrome
ANOVA: Analysis of variance
APPROACH: Alberta Provincial Program for Outcome Assessment in Coronary Heart disease
CABG: Coronary artery bypass surgery
MI: Myocardial infarction
NSTEMI: Non-ST elevation myocardial infarction
PCI: Percutaneous coronary intervention
STEMI: ST elevation myocardial infarction
UA: Unstable angina

## References

[1] Mehta SR, Cannon CP, Fox KA, Wallentin L, Boden WE, Spacek R, Widimsky P, McCullough PA, Hunt D, Braunwald E, Jusuf S (2005). Routine vs. selective invasive strategies in patients with acute coronary syndromes- a collaborative meta-analysis of randomized trials. JAMA.

[2] Bhatt DL, Roe MT, Peterson ED, Li Y, Chen AY, Harrington RA, Greenbaum AB, Berger PB, Cannon CP, Cohen DJ, Gibson CM, Saucedo JF, Kleiman NS, Hochman JS, Boden WE, Brindis RG, Peacock WF, Smith SC, Pollack CV, Gibler WB, Ohman EM (2004). Utilization of early invasive management strategies for high-risk patients with non–ST-segment elevation acute coronary syndromes: results from the CRUSADE Quality Improvement Initiative. JAMA.

[3] Anderson JL, Adams CD, Antman EM, Bridges CR, Califf RM, Casey DE, Chavey WE, Fesmire FM, Hochman JS, Levin TN, Lincoff AM, Peterson ED, Theroux P, Wenger NK, Wright RS (2007). ACC/AHA 2007 guidelines for the management of patients with unstable angina/non–ST-elevation myocardial infarction: executive summary: a report of the American College of Cardiology/American Heart Association Task Force on Practice Guidelines (Writing Committee to Revise the 2002 Guidelines for the Management of Patients With Unstable Angina/Non–ST-Elevation Myocardial Infarction). Circulation.

[4] Camm J, Gray H, Antoniou S, Cadman J, Crowe E, De belder M, Diaz J, Geldard D, Gulhane L, Henderson R, Jahangiri M, Krause T, Lovibond K, Maxwell G, Morris F, Roebuck A, Sloan N, Turner C, Underwood SR, Whitbread M (2010). Unstable angina and NSTEMI: the early management of unstable angina and non-ST segment elevation myocardial infarction. NICE clinical guideline.

[5] Chew D, Anderson F, Avezum A, Eagle K, Fitzgerald G, Gore J, Dedrick R, Brieger D (2010). Six-month survival benefits associated with clinical guideline recommendations in acute coronary syndromes. Heart.

[6] Hamm C, Bassand JP, Agewall S, Bax J, Boersma E, Bueno H, Caso P, Dudek D, Gielen S, Huber K, Ohman M, Petrie M, Sonntag F, Uva MS, Storey R, Wijns W, Zahger D (2011). ESC Guidelines for the management of acute coronary syndromes in patients presenting without persistent ST-segment elevation. Eur Heart J.

[7] Hvelplund A, Galatius S, Madsen M, Rasmussen J, Sørensen R, Fosbøl E, Madsen J, Rasmussen S, Jørgensen E, Thuesen L, Møller C, Abildstrøm S (2011). Influence of distance from home to invasive centre on invasive treatment after acute coronary syndrome:a nationwide study of 24 910 patients. Heart.

[8] Ferreira-Gonzalez I, Permanyer-Miralda G, Heras M, Cunat J, Civeira E, Aros F, Rodriguez J, Sanchez P, Marsal J, Ribera A, Marrugat J, Bueno H (2008). Patterns of use and effectiveness of early invasive strategy in non–ST-segment elevation acute coronary syndromes: an assessment by propensity score. Am Heart J.

[9] Roe MT, Chen AY, Delong ER, Boden WE, Calvin JE, Cairns CB, Smith SC, Pollack CV, Brindis RG, Califf RM, Gibler WB, Ohman EM, Peterson ED (2008). Patterns of transfer for patients with non–ST-segment elevation acute coronary syndrome from community to tertiary care hospitals. Am Heart J.

[10] Ghali WA, Knudston ML (2000). Overview of the Alberta provincial project for outcome assessment in coronary heart disease. Can J Cardiol.

[11] Faris P, Ghali W, Brant R, Norris C, Galbraith D, Knudtson M, for the APPROACH Investigators (2002). Multiple imputation versus data enhancement for dealing with missing data in observational health care outcomes analyses. J Clin Epidemiol.

[12] Khot UN, Johnson ML, Ramsey C, Khot MB, Todd R, Shaikh SR, Berg WJ (2007). Emergency department physician activation of the catheterization laboratory and immediate transfer to an immediately available catheterization laboratory reduce door to balloon time in ST elevation myocardial infarction. Circulation.

[13] LeMay MR, Davies RF, Dionne R, Maloney J, Trickett J, So D, Ha A, Sherrard H, Glover C, Marquis JF, O’Brien ER, Stiell IG, Poirier P, labinaz M (2006). Comparison of early mortality of paramedic diagnosed ST segment elevation myocardial infarction with immediate transport to a designated primary percutaneous coronary intervention center to that of similar patients transported to the nearest hospital. Am J Cardiol.

[14] de Villiers JS, Anderson T, McMeekin JD, Leung RCM, Traboulsi M (2007). Expedited transfer for primary percutaneous coronary intervention: a program evaluation. Can Med Ass J.

[15] O’Gara P, Kushner F, Ascheim D, Casey D, Chung M, de Lemos J, Ettinger S, Fang J, Fesmire F, Franklin B, Granger C, Krumholz H, Linderbaum J, Morrow D, Newby L, Ornato J, Ou N, Radford M, Tamis-Holland J, Tommaso C, Tracy C, Woo Y, Zhao D (2013). 2013 ACCF/AHA guideline for the management of ST-Elevation myocardial infarction. J Am Coll Cardiol.

[16] Alberts M, Latchaw R, Selman W, Shephard T, Hadley M, Brass L, Koroshetz W, Marler J, Booss J, Zorowitz R, Croft J, Magnis E, Mulligan D, Jagoda A, O’Connor R, Cawley C, Connors J, Rose-DeRenzy J, Emr M, Warren M, Walker M (2005). Recommendations for comprehensive stroke centers: a consensus statement from the Brain Attack Coalition. Stroke.

[17] Shultis W, Graff R, Chamie C, Hart C, Louangketh P, McNamara M, Okon N, Tirschwell D (2010). Striking rural-ruban disparities observed in acute stroke care capacity and services in the pacific northwest:implications and recommendation. Stroke.

[18] Gropen T, Magdon-Ismail Z, Day D, Melluzzo S, Schwamm L (2009). Regional implementation of the stroke systems of care model: recommendations of the northeast cerebrovascular consortium. Stroke.

[19] Walters DL, Aroney CN, Chew DP, Bungey L, Coverdale SG, Allan R, Brieger D (2008). Variations in the application of cardiac care in Australia. results from a prospective audit of the treatment of patients presenting with chest pain. Med J Aust.

[20] Wennberg JE, Birkmeyer JD (1990). Appendix on the geography of health care in the United States. The Dartmouth Atlas of Health Care in the United States.

[21] Hassan A, Pearce N, Mathers J, Veugelers P, Hirsch G, Cox J, on behalf of the Improving Cardiovascular Outcomes in Nova Scotia (ICONS) Investigators (2009). The effect of place of residence on access to invasive cardiac services following acute myocardial infarction. Can J Cardiol.

[22] Ellis C, Devlin G, Elliot J, Matsis P, Williams M, Gamble G, Hamer A, Richards M, White H, New Zealand Acute Coronary Syndrome (NZACS) Audit Group (2010). Acute coronary syndrome patients in New Zealand experience significant delays to access cardiac investigation and revascularization treatment especially when admitted to non-interventional centres: results of the second comprehensive national audit of ACS patients. NZ Med J.

[23] Wallentin L, Swahn E, Kontny F, Husted S, Lagerqvist B, Ståhle E (1999). Invasive compared with non-invasive treatment in unstable coronary-artery disease: FRISC II prospective randomised multicentre study. Fragmin and fast revascularization during instability in coronary artery disease investigators. Lancet.

[24] Cannon CP, Weintraub WS, Demopoulos LA, Vicari R, Frey MJ, Lakkis N, Neumann FJ, Robertson DH, DeLucca PT, DiBattiste PM, Gibson CM, Braunwald E (2001). Comparison of early invasive and conservative strategies in patients with unstable coronary syndromes treated with the glycoprotein IIb/IIIa inhibitor tirofiban. N Engl J Med.

[25] Fox KA, Poole-Wilson PA, Henderson RA, Clayton TC, Chamberlain DA, Shaw TR, Wheatley DJ, Pocock SJ (2002). Interventional versus conservative treatment for patients with unstable angina or non-ST-elevation myocardial infarction: the British Heart Foundation RITA 3 randomised trial. Lancet.

[26] Puymirat E, Taldir G, Aissaoui N, Lemesle G, Lorgis L, Cuisset T, Bourlard P, Maillier B, Ducrocq G, Ferrieres J, Simon T, Danchin N (2012). Use of invasive strategy in Non-ST segment elevation myocardial infarction is a major determinant of improved long term survival. J Am Coll Cardiol Int.

[27] O’Brien E, Subherwal S, Roe MT, Holmes DN, Thomas L, Alexander KP, Wang TY, Peterson ED (2014). Do patients treated at academic hospitals have better longitudinal outcomes after admission for non–ST-elevation myocardial infarction?. Am Heart J.

